# Rational High-Throughput System for Screening Emodin High-Yielding Mutant from Marine Strains of *Aspergillus flavipes* HN4-13

**DOI:** 10.3390/md23040174

**Published:** 2025-04-16

**Authors:** Lizhi Gong, Zixuan Li, Meina Xu, Yushan Zhou, Wenqing Zhang, Jian Zhao, Xiujuan Xin, Faliang An

**Affiliations:** 1 State Key Laboratory of Bioreactor Engineering, East China University of Science and Technology, 130 Mei-Long Road, Shanghai 200237, China; y12223076@mail.ecust.edu.cn (L.G.); zxuan-li@163.com (Z.L.); 23012176@mail.ecust.edu.cn (Y.Z.); 23012171@mail.ecust.edu.cn (W.Z.); zhaojian@ecust.edu.cn (J.Z.); 2 College of Pharmacy, Jinan University, Guangzhou 510632, China; meina_0215@163.com; 3 Marine Biomedical Science and Technology Innovation Platform of Lin-gang Special Area, No.4, Lane 218, Haiji Sixth Road, Shanghai 201306, China

**Keywords:** marine strain *Aspergillus flavipes* HN4-13, emodin, ARTP, high-throughput screening, medium optimize

## Abstract

Emodin is an anthraquinone compound known for its diverse biological activities, including anti-tumor and anti-inflammatory effects, making it highly applicable in the fields of biology and medicine. The production of emodin using microorganisms represents a sustainable and environmentally friendly approach. A marine-derived *Aspergillus flavipes* HN4-13 was found to produce emodin, but the yield was too low for industrial production. To develop a high-yielding emodin-producing strain, we established the high-through detection and screening methods of alkaline coloration and deep-well plant culture, enabling the effective selection of high-yielding strains. Following ARTP mutagenesis of the wild strain *A. flavipes* HN4-13, the resulting mutant strain, M1440, exhibited an increased emodin yield of 124.6 ± 4.95 mg/L. Furthermore, the production of the emodin was enhanced by the exogenous addition of metal ions Mn^2+^ to the medium. Specifically, the addition of 3 mM Mn^2+^ resulted in a 133.2% increase in emodin production, with the highest yield reaching 178.6 ± 7.80 mg/L.

## 1. Introduction

Emodin, 1,3,8-trihydroxy-6-methylanthraquinone, is a key compound extracted from *Reynoutria japonica* Houtt and *Rheum palmatum* L. and is known for its broad medicinal value. Emodin is recognized as a protein tyrosine kinase inhibitor and an effective anticancer agent against various tumor cell types [1]. Additionally, it exhibits a wide range of biological activities [2] including anti-inflammatory [3], antiviral [4], neuroprotective [5], antibacterial [6], and hepatoprotective [7] effects. Furthermore, emodin is widely used in healthcare and cosmetics, such as in shampoo and skincare products, and even serves as a natural pigment in dyes. Due to its multiple hydroxyl groups, emodin possesses laxative properties, which make it useful in laxative formulations [8].

The main source of emodin is through extraction from traditional Chinese medicine plants, particularly *Rheum palmatum*. However, the long planting cycle, dependence on uncontrolled season factors, and the traditional production areas located in alpine and high-altitude areas lead to the higher price for the compound. The chemical synthesis of emodin typically requires m-cresol and 3,5-dinitrophthalic anhydride as raw materials to construct the anthraquinone host through two Friedel–Crafts reactions [9], followed by four additional steps of nitro reduction and diazotization. However, the raw materials are uncommon and the yield is low [10,11], leading to a high cost of the chemical synthesis, which is not suitable for industrial scale-up production.

With the development of synthetic biology, the microbial fermentation process has emerged as a viable alternative for emodin production [12,13]. Emodin has been identified as a secondary metabolite in some fungi, including those from the *Aspergillus* and *Penicillium* genera [14]. For instance, in *A. ochra*, after the optimization of fermentation conditions, the emodin yield reached 1.453 mg/L at a preparative scale, demonstrating the potential for biological production of emodin. Furthermore, the unique marine environment, known for its rich biodiversity, provides a valuable resource for the development of novel natural products, yielding many compounds with unique structures and diverse bioactivities [15,16,17]. *Aspergillus flavipes* HN4-13, isolated from the ocean, was previously identified as a producer of emodin through the polyketide pathway [18,19]. During the fermentation process, it was found that the another fermentation product, questin, could be converted into emodin by heating and refluxing in the presence of HBr in an acidic environment [18]. As a result, the yield of questin was included in the total emodin production. After optimizing fermentation conditions and culture medium components, the emodin yield reached 185.56 ± 4.39 mg/L. However, due to genetic instability, the yield of emodin was only 76.6 ± 0.93 mg/L in subsequent repeated experiments. Therefore, to meet industrial production needs, it is essential to obtain a high-yield, genetically stable emodin-producing strain.

In order to acquire high-yield emodin-producing strains, atmospheric and room-temperature plasma (ARTP) has recently been employed for microbial strain improvement. ARTP operates on the principle of atmospheric pressure glow discharge to generate high-energy plasma at room temperature and pressure [20,21]. Plasma-enriched chemically active particles cause intense genetic damage to organisms, and the surviving organisms initiate their endogenous SOS repair system to mitigate the lethal damage. Compared with traditional mutagenesis methods, ARTP mutagenesis has the advantages of a high positive mutation rate, a wide mutation range, mild conditions, and environmental friendliness [22]. ARTP mutagenesis has been widely applied in microbial strain improvement. For example, in the mutagenesis of *Streptomyces albicans* [23], ARTP mutagenesis significantly enhanced ε-polylysine (ε-PL) production in the mutant strain ASR14-116.

The optimum detection measures were the restrictive step for mutant selection, and the simpler the selection methods, the more convenient it is to obtain target strains. High-throughput screening technology is a method that enables the rapid and efficient evaluation of the effects of numerous compounds, biomolecules, or other reagents on specific biological targets, cells, or biological processes. HPLC was the common method for emodin detection on C_18_ column with the elution phase of methanol and 0.1% acetic acid (4:1, *v*/*v*) under 430 nm or 290 nm detector [24]. This method is precise for emodin content measurement, but the procedure is complicated; it not suitable for high-throughput screening within the numerous mutant libraries. It has been reported that hydroxyquinones undergo a color change and darkening in alkaline solutions, a phenomenon known as the Borntrager reaction [25]. When emodin is mixed with an alkaline solution, the reaction produces red or pink colors.

Therefore, on the basis of the selected emodin production strain *A. flavipes* HN4-13, this study further improves the fermentation yield of emodin through strain breeding and a high-throughput screening method of rapid chromogenic detection; following the exogenous substances addition optimization, the mutant strain has an emodin yield of 178.6 ± 7.80 mg/L, which increased the production of emodin by 133.2% compared with the origin strain and medium.

## 2. Results and Discussion

### 2.1. High-Throughput Detection Emodin Production Method

**Figure 1:**
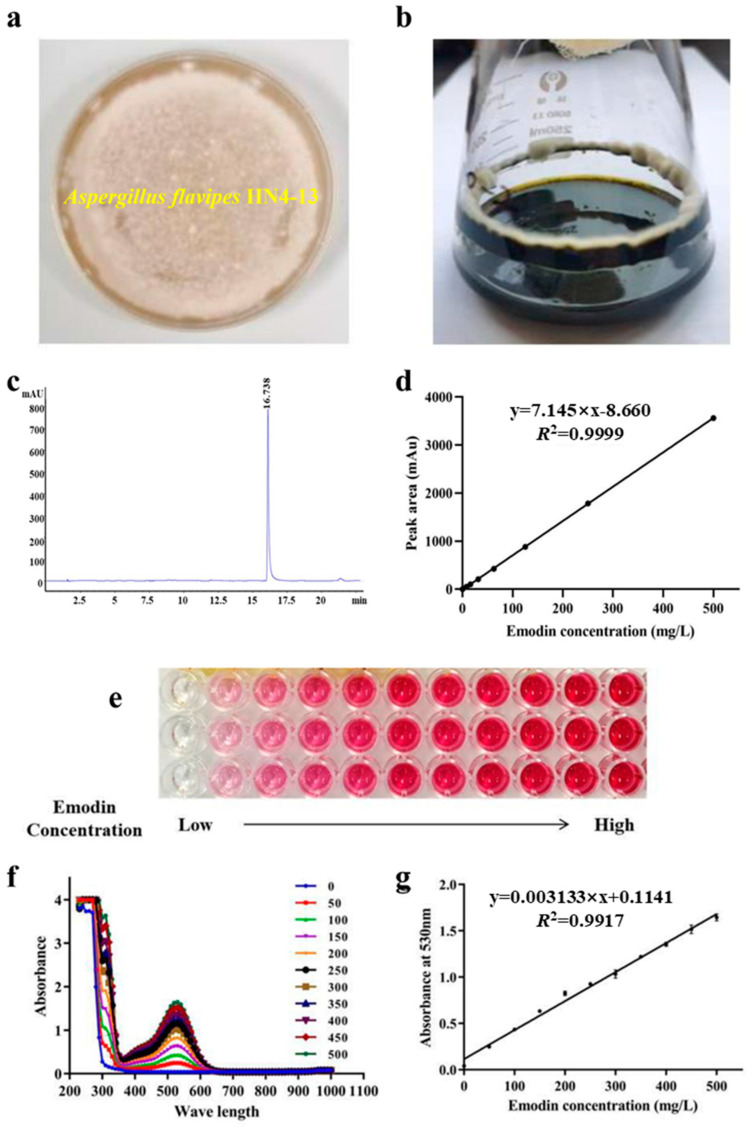
Convenience methods for emodin detection. (**a**) Morphology of *A. flavipes* HN4-13 on solid plate medium. (**b**) Growth of *A. flavipes* HN4-13 in shaker liquid fermentation. (**c**) HPLC chromatogram of emodin. (**d**) Standard curve for emodin concentration determination based on HPLC analysis. (**e**) Color variation of emodin standard solutions (0–500 mg/L) under alkaline conditions, showing red in a 96-well plate. (**f**) Full-wavelength absorbance scan (230–1000 nm) of emodin standard solution. (**g**) Standard curve of emodin absorbance at 530 nm.

Generally, organisms that exhibit vigorous growth on culture media tend to produce higher metabolite yields [26]. In this study, the target strain was inoculated onto solid medium at 28 °C, forming white mycelia, indicating stable growth under these conditions (Figure 1a). Additionally, at 32 °C and 180 rpm, the strain exhibited robust growth in sterile liquid medium (Figure 1b). Notably, as the culture progressed, the medium gradually darkened and turned green, suggesting enhanced secondary metabolism and the increased accumulation of metabolites such as emodin. To accurately quantify emodin in the fermentation broth, we extracted it with ethyl acetate, followed by quantitative analysis using high-performance liquid chromatography (HPLC). Specifically, at a detection wavelength of 254 nm, with 0.1% formic acid (*v*/*v*) methanol solution as the mobile phase, the characteristic elution peak of emodin was observed at 16.5 min (Figure 1c). Additionally, a standard curve for emodin was constructed based on the peak area obtained from HPLC analysis for subsequent quantification (Figure 1d).

Additionally, in an alkaline environment, increasing emodin concentration gradually deepened the red color of its standard solution in a 96-well plate, consistent with the classic Borntrager reaction (Figure 1e) [25]. Given that the color change is closely related to emodin concentration, we hypothesized that its light absorption characteristics could serve as a rapid detection method for high-throughput screening. Therefore, full-wavelength scanning (230–1000 nm, step: 10 nm) of emodin standard samples under basic conditions revealed that absorbance at 530 nm was proportional to emodin concentration (Figure 1f). The linear relationship between emodin concentration and absorbance at 530 nm was determined, as described by the following equation (Figure 1g):

Y = 0.003133X + 0.1141 (*R*^2^ = 0.9917)

This method yielded a linear coefficient of *R*^2^ > 0.99, indicating that this method could be applied to the high-throughput screening of the emodin-producing strains.

### 2.2. Screening the Optimum Fermentation Conditions for Emodin Product Strains

ARTP mutagenesis has generated a large number of mutation libraries, making it necessary to improve screening efficiency and explore high-throughput screening methods. Deep-well plate culture is a widely used technique for high-throughput screening, though different microorganisms require specific growth conditions in deep-well plates [27]. Therefore, prior to ARTP mutagenesis, we optimized deep-well plate fermentation conditions for *A. flavipes* HN4-13 to enhance emodin production. The liquid-to-volume ratio is a critical factor influencing mass transfer in the culture medium [28]. In 48-well plates, the typical filling volume is approximately 1 mL; insufficient liquid leads to excessive evaporation, while excessive liquid increases the risk of cross-contamination between adjacent wells and impairs oxygen transfer [29]. To determine the optimal volume, four different liquid volumes (0.8 mL, 1.0 mL, 1.2 mL, and 1.4 mL) were tested. As an aerobic fungus, *A. flavipes* HN4-13 tends to form spherical aggregates during cultivation, limiting oxygen diffusion to internal cells and potentially restricting secondary metabolite production. To mitigate this issue and enhance oxygen mass transfer, glass beads were added to the culture to prevent microbial aggregation. Two experimental groups—with and without glass beads—were established to assess oxygen transfer efficiency across all four tested liquid volumes.

**Figure 2:**
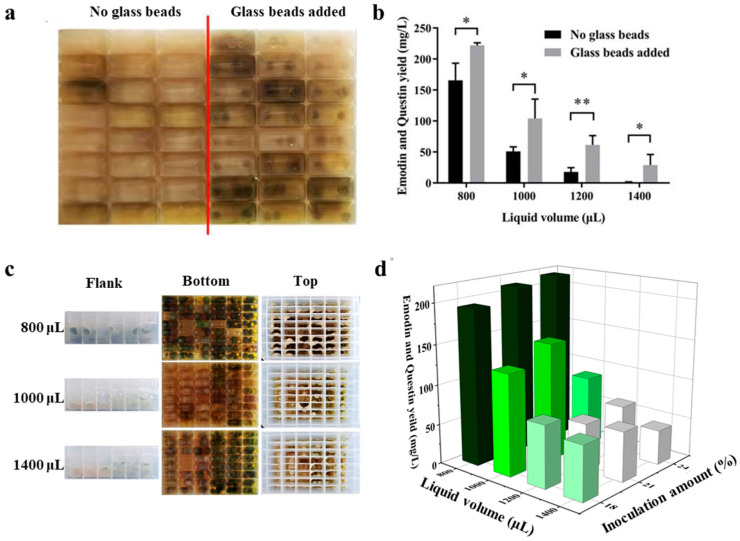
Optimization of fermentation conditions for high yield emodin in 48-well plate. (**a**) Comparison of fermentation broth color with and without glass beads addition (right to left) observed at the bottom of the 48 deep-well plate. (**b**) The statistical analysis of emodin and questin production under different liquid volumes with or without glass beads (* *p* < 0.05, ** *p* < 0.01). (**c**) Growth state of the fermentation broth observed from the flank, bottom, and top of the 48-deep-well under different medium filling volumes. (Glass beads were not added in the left three columns of each plate but were added in the right three columns). (**d**) Effects of liquid volume and inoculum size on emodin and questin yield.

Based on fermentation behavior observed in shake flasks, emodin accumulation leads to a gradual color change in the fermentation broth from light yellow to yellow-green. The experimental results demonstrated that cultures supplemented with glass beads (right side of Figure 2a) exhibited a more intense yellow-green coloration than those without (left side of Figure 2a), indicating that glass beads facilitated *A. flavipes* HN4-13 growth in 48-well plates and promoted emodin accumulation. The high-throughput quantification of emodin in the fermentation broth further confirmed that glass bead addition significantly increased the yields of emodin and its related metabolite, questin (Figure 2b), likely due to the enhanced shear stress mediated by glass beads [29]. Moreover, the highest emodin accumulation was observed at a fermentation volume of 800 μL. Observations from the side, bottom, and top views of the deep-well plates (Figure 2c) revealed that fungal growth was more vigorous in wells containing glass beads (right three columns in Figure 2c) than in those without (left three columns in Figure 2c) at all tested volumes. Notably, the most abundant mycelium formation occurred in the 800 μL fermentation group, suggesting that this volume provides the most favorable fermentation conditions.

The inoculum volume is another key parameter influencing fermentation efficiency [30]. In shake flask fermentation, the conventional inoculation volume for *A. flavipes* HN4-13 is 18%. To optimize this parameter for deep-well plates, three inoculation volumes (18%, 21%, and 24%) were tested. The experimental results showed that at 800 μL fermentation volume and 24% inoculation, the total yield of emodin and questin was highest, reaching 214.9 mg/L (Figure 2d). This increase may be attributed to the regulatory effects of inoculum size on the metabolic state of the fermentation system [31]. In summary, the optimal fermentation conditions for *A. flavipes* HN4-13 in 48-well plates were determined to be 800 μL fermentation volume, 24% inoculation, and the addition of two glass beads. These optimized conditions will be applied for the high-throughput screening of ARTP mutant strains to further improve emodin production efficiency.

### 2.3. ARTP Mutagenesis Parameters and High-Through Selection

**Figure 3:**
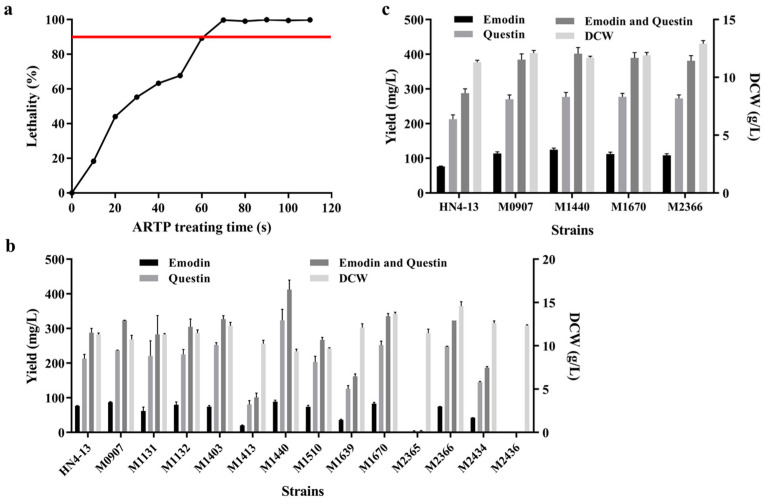
ARTP mutagenic lethal curve and emodin high-yield strain selection results. (**a**) ARTP mutagenic lethal curve showing the relationship between treatment time and lethality rate. (**b**) Shake flask yields of 13 mutants with the highest yields selected from the primary screening. (**c**) Confirmation of yields and dry cell weight (DCW) for four emodin high-yielding strains.

Under fixed conditions of mutagenic power, helium flow rate, and irradiation distance, the mutagenic effect of ARTP treatment on the organism increased with prolonged exposure time, demonstrating a positive correlation between lethality rate (RL) and mutation rate (RM). Previous studies have indicated that when the RL approaches 90%, the positive mutation rate (RP) is high while maintaining low selection pressure on the strain [32,33]. Therefore, under a mutagenesis power of 120 W, helium flow rate of 12 standard liter per minute (SLM), and irradiation distance of 2 mm, the spore suspension was subjected to ARTP mutagenesis for 0–110 s. Following gradient dilution, the treated spores were plated onto solid culture medium, and the corresponding lethality rate was calculated. The results revealed that a 60 s ARTP treatment resulted in an 89.3% RL, aligning with the expected optimal mutation rate (Figure 3a). Consequently, 60 s was selected as the optimal mutation duration, and high-yield emodin-producing strains were screened using the previously established high-throughput screening method.

After nine rounds of deep-well plate screening, a total of 3069 mutants were obtained from the ARTP mutation library. Under conditions where the RL was 89.3%, the RM was calculated to be 84.0%, with an RP of 58.7%. The screening standard was based on an increase in emodin yield of more than 5% compared to the untreated group. These strains were subsequently re-screened in shake flask fermentation. Since questin, a byproduct of *A. flavipes* HN4-13 fermentation, can be converted into emodin via demethylation, its yield was included in the total emodin output. Additionally, dry cell weight (DCW) was measured as an indicator of strain growth. As shown in Figure 3b, 13 mutants exhibited significantly increased emodin and questin production.

Considering the combined yields of emodin and questin, four promising mutants—M0907, M1440, M1670, and M2366—were selected for further shake flask fermentation analysis. Specific yield data are presented in Figure 3c and Table 1. The results demonstrated that, compared to the wild-type strain, these four mutants exhibited varying degrees of improvement in emodin and questin production. Therefore, further studies will be conducted to evaluate the genetic stability of these mutants.

### 2.4. Genetic Stability Analysis of Four Emodin High-Yielding Mutants

**Figure 4:**
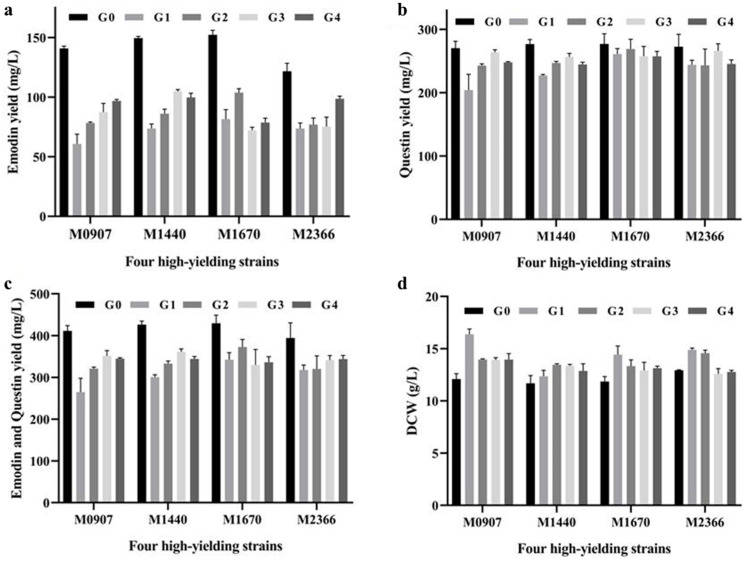
Genetic stability analysis of four high-yielding strains. (**a**) Emodin yield. (**b**) Questin yield. (**c**) Combined yield of emodin and questin. (**d**) Dry cell weight (DCW).

To assess the genetic stability of high-yielding strains, four high-yield mutants were sub-cultured for four consecutive passages. After the first passage (G1), a significant decline in emodin production was observed in these mutants (Figure 4a), indicating genetic instability in high-yield traits during sub-culture. Although partial recovery was noted in subsequent passages, the initial decrease suggests that the stability of the high-yield phenotype requires further investigation. Similarly, questin production also declined across successive subcultures (Figure 4c). In contrast, the dry cell weight (DCW) of the four high-yield mutants increased (Figure 4b,d), suggesting that while the growth rate of the mutants improved, the biosynthesis of secondary metabolites was adversely affected. Since the genetic background of the wild-type strain remains incompletely characterized, this phenomenon highlights the need for further studies to enhance the genetic stability of high-yield traits. To achieve this, secondary or even multiple rounds of mutagenesis could be considered. Additionally, previous studies suggest that the further a mutant’s genetic background deviates from the wild-type strain, the more likely it is to maintain its high-yield characteristics [34].

### 2.5. Morphological Observation of Hyphae

**Figure 5:**
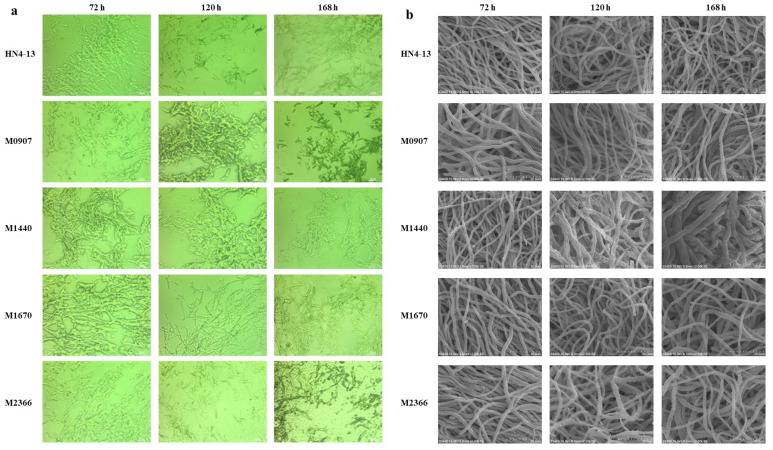
The Morphological observation of emodin product-producing hyphae. (**a**) Cryosection images of 4 high-yielding mutant strains at 72 h, 120 h, and 168 h. (**b**) SEM images (2000×) of 4 high-yielding mutant strains at 72 h, 120 h and 168 h.

To study the mycelial morphology of high-yield and low-production emodin strains, three key fermentation time points of 72 h, 120 h, and 168 h were selected. These time points correspond to the rapid mycelial growth phase, the peak emodin accumulation phase, and the end of fermentation, respectively. Mycelial morphology is closely correlated with biomass [35]. Cryosection observations of mutant mycelia are shown in Figure 5a. The wild-type strain HN4-13 exhibited thinner mycelia and produced more spores at the end of fermentation. In contrast, the mycelia of mutants M0907 and M2366 were more finely divided and morphologically similar to the wild-type strain. Mutant strains M1440 and M1670 displayed thicker mycelia at 168 h while maintaining similar initial mycelial lengths, suggesting improved growth conditions compared to the other strains [18]. The emodin production data further supported this inference, with M1440 and M1670 demonstrating superior growth characteristics.

The mycelia of wild-type strain HN4-13 remained slender and stable across all three time points (72 h, 120 h, and 168 h). In contrast, the morphology of mutants M0907 and M1670 became increasingly heterogeneous over time, with coexisting mycelia of varying thicknesses. The mycelia of M1440 exhibited progressive thickening at each time point, while M2366 demonstrated pronounced thickening at 168 h compared to 72 h, as shown in Figure 5b. Scanning electron microscopy (SEM) analysis confirmed that none of the four high-yielding mutant strains exhibited rough or bifurcated surfaces, indicating the absence of significant stress or damage during growth. The inconsistent mycelial thickness observed in M0907, M1670, and M2366 may reflect different growth stages of the cells. Notably, M1440 exhibited a consistent increase in mycelial thickness following mutagenesis, with pronounced morphological changes over fermentation time.

Considering emodin production, genetic stability, and mycelial morphology, M1440 and M1670 achieved the highest overall performance indices, while M0907 and M2366 exhibited slightly lower values. M1440 exhibited thicker and more stable mycelium than M1670, establishing it as the optimal emodin-producing mutant strain from ARTP mutagenesis. M1440 produced 124.6 ± 4.95 mg/L of emodin, with questin yield reaching 276.9 ± 13.03 mg/L, resulting in a total metabolite yield of 401.5 ± 17.98 mg/L. Future research will focus on optimizing emodin fermentation in M1440 by investigating the effects of exogenous substances such as amino acids, polyketide pathway precursors, and surfactants, as well as scaling up fermentation in a 5 L bioreactor.

### 2.6. Effect of Metal Ion Addition on Emodin Production

**Figure 6:**
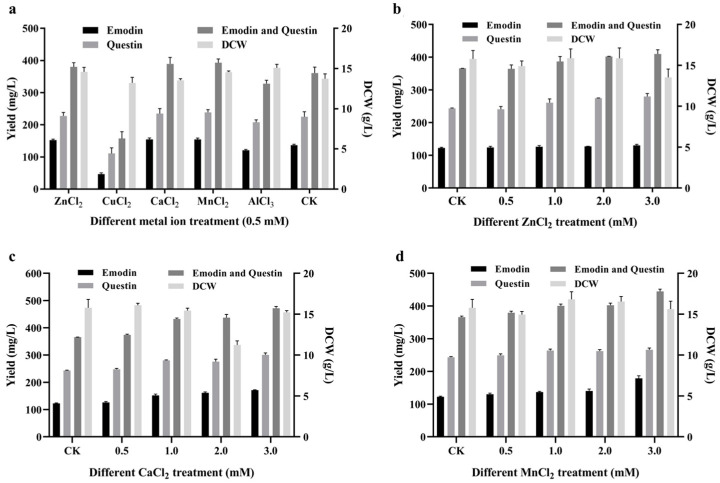
Effects of metal ion at different concentrations on emodin production. (**a**) Effects of various metal ion on emodin production. (**b**) Effects of different concentrations of ZnCl_2_ additions on emodin production. (**c**) Effects of different concentrations of CuCl_2_ additions on emodin production. (**d**) Effects of different concentrations of MnCl_2_ additions on emodin production.

The supplementing of metal ions into the growth medium is a proven strategy for enhancing the production of fermentation [36]. As shown in Figure 6a, the addition of 0.5 mM of five different metal ions at 48 h of fermentation resulted in similar DCWs compared to controls, indicating that 0.5 mM metal ions did not significantly affect fungal growth. The addition of CuCl_2_ significantly reduced emodin yield, while AlCl_3_ reduced the total yield of both questin and emodin by 12.0%. Therefore, these two metal ions are not conducive to emodin synthesis. Conversely, the addition of ZnCl_2_, CuCl_2_, and MnCl_2_ stimulated the production of both emodin and questin, warranting further exploration of the effects of varying concentrations of these three metal salts on emodin synthesis.

The effects of Zn^2+^, Ca^2+^, and Mn^2+^ (0.5–3.0 mM) on the synthesis of emodin and questin were subsequently evaluated. The results showed that the addition of these metal ions enhanced emodin and questin production in a concentration-dependent manner. Specifically, 3 mM Zn^2+^ increased emodin production by 6.2%, reaching 130.0 ± 3.57 mg/L; 3 mM Ca^2+^ increased emodin production by 39.6%, reaching 171.0 ± 1.36 mg/L; and 3 mM Mn^2+^ increased emodin production by 45.8%, reaching 178.6 ± 7.80 mg/L, as shown in Figure 6b–d. These findings indicate that 3 mM Mn^2+^ had the most pronounced effect on promoting emodin synthesis

## 3. Materials and Methods

### 3.1. Strain and Culture Conditions

*A. flavipes* HN4-13 (CCTCC No. AF2, 015, 022) was provided by Professor Renxiang Tan from Nanjing University of Traditional Chinese Medicine.

The spores (from wild type strain, *A. flavipes* HN4-13) used for ARTP mutagenesis and the mutants generated by ARTP mutagenesis were grown on solid medium, which contains 40 g/L glucose, 10 g/L peptone, 30 g/L sea salt, and 25 g/L agar, at 28 °C after being sterilized at 115 °C for 30 min. After the selection of the high-yielding mutants, sterility medium containing soluble starch 59.3 g/L, yeast extract 10 g/L, sea salt 30 g/L, KH_2_PO_4_ 10 g/L, MgSO_4_•7H_2_O 0.05 g/L, and FeSO_4_•7H_2_O 0.01 g/L was used for 7 d of emodin fermentation culture at 32 °C and 180 r/min [18].

### 3.2. Fermentation Product Extraction Method and DCW Detection

#### 3.2.1. Emodin Quantification Using HPLC

The detection method for emodin production was established by HPLC. The gradient concentrations of emodin in methanol were set as 7.8125, 15.625, 31.25, 62.5, 125, 250, and 500 mg/L. Then, all samples were filtered through a 0.22 μm membrane and analyzed by HPLC at 310 nm using C-18 Column (4.6 mm × 250 mm) with the column temperature of 30 °C, methanol with 0.1% Formic acid (*v*/*v*) as mobile phase, and 0.8 mL/min flawing rate.

#### 3.2.2. Extraction Method of Emodin from Fermentation

After fermentation, ethyl acetate was added to extract the emodin in the fermentation broth. After sonication for 30 min following shaking for 30 min, and soaking overnight, the ethyl acetate layer containing emodin was collected. The above steps were repeated three times; all collected ethyl acetate was vaporized under pressure, re-dissolved in methanol, and detected by HPLC.

#### 3.2.3. Biomass Analysis of the Fermentation

After fermentation, the broth was filtered off with a negative pressure Buchner funnel, and the filter paper with the fungi was placed in an electric heating constant temperature blast drying oven at 50 °C to dry for more than 24 h, until the filter paper was dried and its weight was not changed. The filter was weighed by an electronic balance, and the difference between the weight of the dried filter paper and the net weight of the filter paper was calculated to obtain the dry weight DCW of the fungi.

### 3.3. Establishment of Emodin with High-Throughput Detection Method

Emodin belongs to the family of anthraquinone, and the anthraquinone compounds appear red in alkaline solution, referred to as the Borntrager reaction. As the production of emodin increases, the reaction color becomes darker. This can be used to indicate the production of emodin during fermentation. If the emodin–alkaline product exhibited distinct absorption, the fermentation broth was scanned under the full wavelength absorbance detector after adding 1M NaOH a 1:1 ratio, placed in a 96-well plate.

### 3.4. Establishment of Emodin-Producing Culture Condition on Well Plate

Before ARTP mutagenesis, we investigated optimal deep-well plate fermentation conditions for *A. flavipes* HN4-13 to produce emodin. The conditions included culture volumes of 800 μL/2 mL, 1000 μL/2 mL, 1200 μL/2 mL, and 1400 μL/2 mL, with or without the addition of glass beads.

### 3.5. ARTP Mutagenesis

The ARTP method was an effective strategy for microorganisms’ evolution, and has been used for its good properties [37]. After scraping the spores from the solid plate into sterile water, glass beads were added, and the mixture was shaken violently to release the spores. The mycelium was filtered out with sterile cotton, and the spore concentration was adjusted to 1 × 10^5^ CFU/mL for ARTP mutagenesis. The spore suspension was dipped onto the sterilized metal plate (5 mm diam). The plate was treated for 0–110 s (every 10 s set a group) under the conditions of 120 W power, 12 SLM (standard liters per minute) helium flow, and 2 mm irradiation distance. The mutagenized spores were diluted and spread on the solid plate. After culturing a clear single colony, micro-fermentation was carried out in a 48-deep-well plate.

The calculation formulas for the lethality rate RL, mutation rate RM, and positive mutation rate RP of ARTP mutagenesis treatment are as follows:

(1) $$Lethality\;Rate\;(R_{L})\;(\%)=\frac{U-T}{U}\times 100\%$$

(2) $$Mutation\;Rate\;(R_{M})\;(\%)=\frac{M}{U}\times 100\%$$

(3) $$Positive\;Mutation\;Rate\;(R_{P})\;(\%)=\frac{P}{U}\times 100\%$$

where *U* is the total number of colonies grown on the solid plate from the spore suspension before ARTP mutagenesis, *T* is the total number of colonies grown on the solid plate from the spore suspension after ARTP mutagenesis, *M* is the number of colonies with changes in emodin production after ARTP mutagenesis compared to the untreated group, and *P* is the total number of colonies with an increase in emodin production after ARTP treatment relative to the untreated group.

### 3.6. Analysis of Genetic Stability of Emodin High-Yielding Mutants

The high-yield characteristics of the high-yield mutants obtained by ARTP can be stably inherited and expressed, which is important for application, for fungi very easily lose some excellent traits during passage [38]. When the mutants enrich some special secondary metabolites, it may change the metabolic pathway due to its sensitivity to the environment during the passage, reduce the yield of some main products, and produce other secondary metabolites and even fungal degeneration. Whether the emodin high-yield traits can be stably inherited needs to be tested by multiple generations.

The screened high-yielding mutants were spore-passaged, that is, the spores produced by the parental generation were spread on the solid medium, and after the abundant mycelium regenerated, the yield of the shake flask was measured, and the next-generation passage was carried out repeatedly for 9 generations.

### 3.7. Mycelial Appearance

Mycelial morphology was observed by frozen section. Collect the mycelia, centrifuge and dry the liquid, put it into an embedding box, add embedding medium, and freeze at −20 °C for 4 h. After the embedding medium is completely solidified, place it in a microtome and slice. Place the normal-temperature glass slide close to the cut slice, and use the temperature difference to make the slice stick to the glass slide; observe and take pictures.

Mycelial morphology was also observed by scanning electron microscope (SEM). The mycelia were collected, washed with PBS, and fixed with 2.5% glutaraldehyde for 2 h in the dark. After the fixative was completely washed with PBS, it was dehydrated step by step with graded ethanol. Finally, the cells dehydrated in absolute ethanol were dried in a carbon dioxide critical drier, and they were observed and photographed with a scanning electron microscope (Hitachi S-3400N, Tokyo, Japan) after gold plating.

### 3.8. Exogenous Metal Ion Addition

It is widely recognized that many key enzymes involved in secondary metabolism require metal ions as cofactors [39]. Consequently, adding certain metal ions may fulfill the cofactor requirements of these enzymes, thereby enhancing their catalytic efficiency. The chlorides of the metal ions Cu^2+^, Ca^2+^, Mn^2+^, and Al^3+^ were prepared as 1 M stock solutions, filtered through a 0.22 μm filter membrane, and added at 0.5‰ after 48 h of fermentation, resulting in a final concentration of 0.5 mM. Since ZnCl_2_ can form a small amount of Zn (OH)_2_ in water, which is insoluble, it was prepared as a 0.25 M stock solution, sterilized by autoclaving, and added after 48 h of fermentation at a concentration of 2‰, achieving a final concentration of 0.5 mM.

## 4. Conclusions

Emodin has significant market potential due to its diverse biological activities, and microbial fermentation presents a feasible and sustainable production strategy. In this study, *A. flavipes* was used as the starting strain, and high-yield mutants producing emodin and questin were successfully screened through a combination of ARTP mutagenesis and exogenous metal ion regulation.

First, an efficient emodin detection system was established using the alkaline chromogenic method for screening high-yield strains. The ARTP mutation conditions were then optimized, and four high-yield emodin mutants were identified from a large pool of ARTP mutants via 48-well plate screening. Based on evaluations of the emodin and questin yield, genetic stability, and mycelial morphology, mutant M1440 was selected as the best strain (Figure 3c), with an emodin yield of 124.6 ± 4.95 mg/L, a quercetin yield of 276.9 ± 13.03 mg/L, and a total combined yield of 401.5 ± 17.98 mg/L (Table 1). Additionally, the supplementation of exogenous Mn^2+^ ions significantly enhanced emodin production. Among the tested concentrations, 3 mM manganese exhibited the most remarkable effect, increasing the emodin yield to 178.6 ± 7.80 mg/L, a 133.2% increase compared to the initial yield. This also elevated the quercetin yield to 266.1 ± 5.30 mg/L, resulting in a total combined yield of 444.7 ± 7.10 mg/L.

In conclusion, the high-yield mutants identified in this study demonstrate considerable potential for industrial-scale emodin fermentation. Further optimization of the culture medium and fermentation process could further enhance production, providing crucial support for the efficient, environmentally friendly, and sustainable large-scale production of emodin.

## Figures and Tables

**Table 1:** Yields of 4 high-yielding mutant strains.

Strains	Emodin (mg/L)	Questin (mg/L)	Sum (mg/L) *^a^*	DCW (g/L)
HN4-13	76.6	212.9	287.5	11.3
M0907	114.1	270.4	384.5	12.1
M1440	124.6	276.9	401.5	11.7
M1670	112.2	277.1	389.3	11.9
M2366	108.5	272.6	381.1	12.9

*^a^* means the sum of emodin and questin yields.

## Data Availability

The generated data are available from the corresponding author and all data are presented in this paper.
